# Occurrence of *Escherichia coli* O157:H7 in cattle feces and contamination of carcass and various contact surfaces in abattoir and butcher shops of Hawassa, Ethiopia

**DOI:** 10.1186/s12866-017-0938-1

**Published:** 2017-01-25

**Authors:** Biruhtesfa Atnafie, Degmawi Paulos, Mesele Abera, Genene Tefera, Dereje Hailu, Surafel Kasaye, Kebede Amenu

**Affiliations:** 1 School of Veterinary Medicine, Hawassa University, P.O. Box 5, Hawassa, Ethiopia; 2 Ethiopian Biodiversity Institute, Addis Ababa, Ethiopia; 3 Present Address: Department of Animal Science, Salale University, P.O. Box 254, Fiche, Ethiopia; 4 Present Address: College of Veterinary Medicine and Agriculture, Addis Ababa University, P.O. Box 34, Bishoftu, Ethiopia

**Keywords:** *E. coli* O157:H7, Abattoir, Antimicrobial susceptibility, Biolog system, Butcher shops

## Abstract

**Background:**

Despite of the sanitation measures in municipal abattoirs to reduce contamination, *Escherichia coli* continues to be a health hazard. The present study was conducted on 150 apparently healthy slaughtered cattle at municipal abattoir and in 50 different butcher shops in Hawassa town, Ethiopia. The objectives of the study were investigating the occurrence and antimicrobial resistance of *E. coli* O157:H7 isolated from fecal samples, carcasses swab, contacts surfaces (swabs of meat handlers hands, knife and clothes of meat transporters) as well as from butcher shops (meat samples, swabs from cutting board swab, butcher men hand and knife surface). *E. coli* O157:H7 was isolated and identified using bacteriological culture, biochemical tests and Biolog identification system. All *E. coli* O157:H7 isolates were then checked for their antimicrobial susceptibility pattern using eleven selected antimicrobial discs.

**Results:**

Of the entire set of 630 samples, 2.4% (15/630) (95% CI = 1.3–3.9%) were positive for *E. coli* O157:H7. When disaggregated by the sources of the samples, *E. coli* O157:H7 were prevalent in 2.8% (11 of 390) of the abattoir samples, of which 4.7% of the fecal sample and 2.7% of the carcass swabs. And *E. coli* O157:H7 were positive in 1.7% (4 of 240) of butcher shop specimens of which 2% of meat sample and 3.3% of Cutting board swabs. No statistically significant difference in the prevalence of *E. coli* 0157: H7 between sex, origin, and breed of cattle. The isolated *E. coli* O157:H7 were found to be100% susceptible to cefotaxime, ceftriaxone, gentamycin, kanamycin and nalidixic acid.

**Conclusion:**

This study concludes the occurrence of *E. coli* O157:H7 and the presence of multiple antibiotic resistance profiles in cattle slaughtered at Hawassa municipal abattoir and retail meat sold at butcher shops. This indicates high risk to public health especially in Ethiopia where many people consume raw or under cooked meat. Regulatory control of antibiotics usage in livestock production and pharmaco-epidemiological surveillance in food animals and animal products is hereby recommended to ensure consumer safety.

## Background

*Escherichia coli* (*E. coli*) are group of bacteria residing in the gastrointestinal tracts of mammals commensally, often without pathogenic effects to the animals. Most *E. coli* strains are also non-pathogenic to humans but detection of *E. coli* in foods intended for human consumption shows poor in hygiene during production, processing or preparation. Ultimately, detection of *E. coli* in food is indicative of fecal contamination and presence of other dangerous pathogenic microorganisms which can compromise the health and wellbeing of consumers. In addition to hygienic indicator, some strains of *E. coli* are directly pathogenic to humans. The best example is shiga toxin-producing *Escherichia coli* O157:H7 (STEC O157) which can cause severe enteric infections. Symptoms of STEC O157 infection may include abdominal pain, bloody diarrhea, hemorrhagic colitis and haemolytic uremic syndrome (HUS) [1, 2]. In this respect, numerous sporadic infections and outbreaks caused by STEC O157 have been reported worldwide in many countries. The majority of STECO157 infections are food borne more specifically associated with cattle sources. Historically, STEC O157 was first linked to outbreaks of severe bloody diarrhea in 1982, and is often referred to as a “recently emerged” human pathogen [3]. Microbiologically culture proven *E. coli* O157 diarrheal cases have been reported from a number of African countries including South Africa, Swaziland, Central African Republic, Kenya, Uganda Gabon, Nigeria and Ivory Coast [4].

In developing countries, including Ethiopia, animals are commonly slaughtered and dressed under unhygienic conditions and this further compromises the microbiological quality and safety of the meat obtained from the animals [29, 30]. This can consequently risk the health of consumers. In the presence of all the above situations, so far there are only few studies which addressed to assess the prevalence and distribution of *E. coli* O157:H7 in humans, animals or in foods of animal origin in Ethiopia [5–8, 30]. Therefore, the objectives of the present study were: (i) to isolate and identify *E. coli* O157:H7 from cattle feces, carcasses and contact surfaces at abattoir and butcher shops, (ii) to determine to what extent the abattoirs and butcher house environments serve as sources of *E. coli* O175:H7 and (iii) to determine the antimicrobial susceptibility pattern of the isolates.

## Results

### Occurrence of *E. coli* O157:H7

Result of the occurrence of *E. coli* O157:H7 is depicted in Table 1. Of the total 630 samples examined, 78 were positive for *E. coli* using selective culture (C-SMAC) and biochemical tests (indole and TSI). Upon further screening using Rainbow agar O157, out of 78 isolates 24 of them were found to be presumptively *E. coli* O157:H7. Finally, confirmation using OmniLog identification system resulted in 15 samples positive for *E. coli* O157:H7 with overall prevalence of 2.4% (95% confidence interval: 1.3–3.9%). When disaggregated according to the types of samples examined, the specimens positive for *E. coli* O157:H7 were: feces (7), carcass swabs (4), meat (3) and cutting board swab (1). There was no statistically significant difference in the occurrence of *E. coli* O157:H7 between butcher houses and abattoir (*p* > 0.05), assuming *E. coli* O157:H7 positive animal when the bacterium is detected only in faecal sample. Though fecal sample is considered as source of carcass contamination, statistically significant difference was not observed (*p* > 0.05).

**Table 1:** Isolation frequency of *E. coli* O157:H7 from different sample types in abattoir and butcher shops, Hawassa, southern Ethiopia

Sample source	Sample type	Number of samples		95% confidence interval
		Examined	Positive (%)	
Abattoir	Fecal sample	150	7 (4.7)	2.0–10.0
	Carcass Swab	150	4 (2.7)	0.4–4.2
	Knife swab	30	0	
	Personnel hand swab	30	0	
	Meat transporters cloth swab	30	0	
Butcher shops	Meat sample	150	3 (2)	4.0–6.0
	Butcher men hand swab	30	0	
	Cutting board swab	30	1 (3.3)	0.08–17
	Knife swab	30	0	
Total		630	15 (2.4)	1.3–3.9

The prevalence of *E. coli* O157:H7 at animal level was 4.7% (7/150) (95% CI [2.0 – 10.0]). Carcass swab of *E. coli* O157: H7 statuses were considered as indicator of contamination and were not used for the calculation of prevalence at animal level (feces). There was no statistically significant difference (*p* > 0.05) in the prevalence of *E. coli* O157:H7 between sex, origin and breed. However, there was statistically significant difference (*p* ≤ 0.05) in the occurrence of *E. coli* O157:H7 between age groups (Table 2), in which higher prevalence was found in age group of ≥ 7 years than younger animals.

**Table 2:** Association of different risk factors with *E. coli* O157:H7 occurrence at animal level

Variable	Total animals examined	Total animals positive (%)	Fisher’s exact test	*p*-value
Sex				
Male	138	7 (4.7)	0.63	1.0
Female	12	0		
Age				
3 years	25	2 (1.3)	4.7	0.04
4–6 years	81	1 (0.7)		
≥ 7 years	44	4 (2.7)		
Origin				
Tula	40	2 (1.3)	5.5	0.36
Arsi Negelle	24	3 (2)		
Wondogenet	7	0		
Hawassa	30	0		
Yirgalem	34	1 (0.7)		
Tikurwoha	13	1 (0.7)		
Breed				
Crossbred	13	0 (0)	0.69	1.0
Local (zebu)	137	7 (4.7)		

### Susceptibility of the isolates to different antimicrobials

**Fig. 1:**
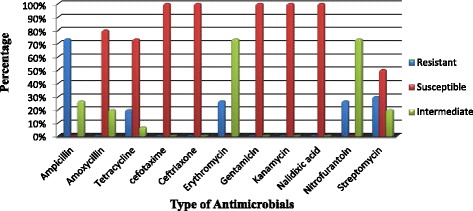
Antimicrobial susceptibility profile of *Escherichia coli* O157:H7 isolated from abattoir and butcher shops in Hawassa

Subjecting of the 15 isolates of *E. coli* O157:H7 to various antimicrobials resulted in different susceptibility patterns. The isolates were found to be 100% susceptible to gentamycin (GEN 10 μg), ceftriaxone (CTR 30 μg), nalidixic acid (NA 30 μg) kanamycin (k 5 μg) and cefotaxime (CTX 30 μg).the result showed a decreased susceptibility pattern of the isolates to amoxicillin-clavulanic acid (AMC 30 μg) (80%), tetracycline (TE 10 μg) (73.7%) and streptomycin (S 10 μg) (50%). The details of susceptibility and resistance pattern of all isolates of *E. coli* O157:H7 are described in Fig. 1 and Table 3, respectively.

**Table 3:** Antimicrobial resistance patterns of *E. coli* O157:H7 isolates

Resistance pattern	No. of isolates	Percent
Ampicillin	4	26.7
Ampicillin, kanamycin	3	20.0
Ampicillin, kanamycin, nitrofurantoin	2	13.3
Ampicillin, kanamycin, tetracycline	1	6.7
Ampicillin, tetracycline, nitrofurantoin	1	6.7
Ampicillin, erythromycin, kanamycin	2	13.3
Ampicillin, tetracycline, erythromycin, kanamycin	1	6.7
Ampicillin, erythromycin, kanamycin, nitrofurantoin	1	6.7
Total	15	100

## Discussion

Human infections of *E. coli* O157:H7 have mostly been recognized to be originated from animal source foods [28]. Domestic ruminants, mainly cattle, sheep, and goats, have been established as major natural reservoirs for STEC and play a significant role in the epidemiology of human infections [9]. In the present study, presence of *E. coli* O157:H7 on carcasses suggests transfer of fecal material onto the carcass during the slaughter process, which may suggest that currently available dressing procedures at the abattoir cannot be reliable to prevent fecal contamination during slaughtering.

Observed prevalence of *E. coli* O157:H7 at abattoir level (2.8%) is in line with the findings from Ethiopia [7], South Africa [10], United Kingdom [11, 12] and Ireland [13] which reported 2.7, 2.8, 2.9, 3.2 and 3.0%, respectively. Compared with the present study, higher prevalence of *E. coli* O157:H7 have been also reported (8% from Ethiopia [5] and 6.4–9.6% from Iran [14–16]. Although the numbers of samples positive for STEC under some categories of the considered risk factors were small to make statistically valid comparisons, more positive animals were found to be male, local breed animals and those in old age groups. The prevalence of *E. coli* O157:H7 showed statistically significant difference (*p* ≤ 0.05) among the different age groups. Observed variation in prevalence among studies could be attributed to difference in sampling and isolation procedures, variability in sampled populations, diverse geographical origins of cattle, numbers of cattle, study design, season, abattoir conditions and treatment with antimicrobial substances during the process [17–19].

The higher occurrence of *E. coli* O157:H7in feces of the slaughtered animals when compared with carcass swabs in the present study is in line with the findings at Manhattan abattoir (4.7%) [20] and in Great Britain (4.7%) [21]. Studies have also showed that prevalence of *E. coli* O157:H7 shed from animal feces can vary significantly in relation to time, age of animals and nature of feeds [18, 22].

In the present study, all of the 15 isolates were found to be entirely susceptible to ceftriaxone (CTR 30 μg), cefotaxime (CTX 30 μg), gentamycin (GEN 10 μg), kanamycin (K, 5 μg) and nalidixic acid (NA30μg). This is in agreement with previous studies from Ethiopia [5, 8]. However, a study conducted in Saudi Arabia revealed that there was resistant strain to nalidixic acid (NA30μg), cefotaxime (CTX 30 μg), and gentamycin (GEN 10 μg) [23]. This variation probably attributed to the expression of resistant gene code by the pathogen which is associated with emerging and re-emerging aspects of the isolates with regards to different agro ecology [24]. On the other hand, in the present study more than half of the isolates were resistant to ampicillin (AMP 10 μg). This might be due to inappropriate or excessive use of antibiotics for therapeutic and prophylactic purpose both for *E. coli* and other infections.

In our present study, we used OmniLog System which is comparable to molecular techniques. OmniLog System use redox (Oxidation reduction) reaction which enables testing of Gram negative and Gram positive bacteria in the same test panel. Actually it would have been good to use a molecular test (like 16sRNA) which is more accurate than Omnilog but this could not be realized due to resource constraints at the time of the study.

## Conclusions

In conclusion, the occurrence of *E. coli* O157:H7 in apparently healthy slaughtered cattle with some antimicrobial resistance pattern suggests a potential risk to public health. The presence of *E. coli* O157:H7in fecal sample followed by carcass swab highly suggests unhygienic practices in the abattoir and the poor hygiene is the key source of microbial contamination of the meat. Our study calls for developing preventive approach to control *E. coli* O157:H7 contamination in meat production chain by imposing strict hygienic meat processing practices in Hawassa municipal abattoir. This can be done by ensuring Good hygienic Practice, Good Manufacturing Practice and if possible Hazard Analysis of Critical Control Points (HACCP) at every stage of the beef supply chain, from the farm, through the abattoir, to the butcher houses, and those involved with the handling and processing. Further investigations using molecular typing should be conducted.

## Methods

### Study area and population

The study was carried out in Hawassa city, located 273 km South of Addis Ababa. Geographically, the City lies between 7°3′ latitude North and 38°28′ longitudes East. The mean annual precipitation is 933.4 mm. Temperatures vary between 5 and 34 °C. The landform is plain with reddish volcano soil. Its population is increasing at an alarming rate. Currently the total population projected to 329, 000 [25]. Samples were collected from November 2014 to April 2015. In Hawassa city municipality abattoir, on average 75 heads of cattle are slaughtered daily for local consumption. No other animal species were slaughtered in the abattoir during the study period. Both sexes and various age groups of cattle were slaughtered during the study period. The cattle slaughtered at Hawassa abattoir were originated from Hawasa itself and different nearby areas, especially Tikurwoha, Tula, Yirgalem, Wondogenet and Arsi Negelle. At the time of the study, the manpower of the abattoir comprised: six meat inspectors, three sanitary, 14 eviscerators, four flayers, four carcass splitters, four cleaners, eight transporters, three management staff, two drivers and two guards.

Similarly, at the time of the study in Hawassa city about 100 butcher shops were legally registered and obtaining carcasses from the municipal abattoir. Informal observations of the butcher shops indicated generally poor hygienic status and normally they didn’t use any disinfectant to clean the wall, floor and cutting board. In addition, the visceral organs were put very near to the carcass when displayed for sale or when meat cut into pieces for selling or consumption. Moreover, it was observed that the cutting boards made from wooden materials were often used.

### Study design and sampling

A cross-sectional study was conducted involving sample collection from apparently healthy slaughtered cattle, abattoir environment and butcher shops. On each sampling day, usually once a week, 7–10 animals were selected by using systematic random sampling method. Before slaughter, information about each animal (age, sex, breed, health status and origin) was recorded using format prepared for this purpose. The types of samples collected from abattoir were fecal contents of the animals, carcass swabs from slaughtered animals, swabs from abattoir environments (eviscerator’s knife, eviscerator hands, transporter clothes). From butchers shops the collected samples included: knife swab, butcher’s hand swab, cutting board swab and meat samples. All the samples for the study were collected under strict aseptic procedures and then transported in ice box to the Veterinary Microbiology Laboratory of the School of Veterinary Medicine of Hawassa University and stored at 4 °C until processed for the presence of *E. coli* O157:H7.

Previous study made in Bishoftu abattoir showed the prevalence of *E. coli* O157: H7 to be 8% in cattle [5]. Therefore, by using this 8% expected prevalence, at a confidence level of 95% and required absolute precision of 5%, the minimum sample size was 114 [26]. However, in order to increase the precision of the study a total of 150 animals were examined. Therefore, 150 carcass swab and 150 fecal samples were collected from 150 slaughtered cattle. Whereas carcasses in contact surface swabs were taken once for each sampling day.

### Isolation of *E. coli* O157:H7

Approximately 1 ml/1 g of feces (homogenized when possible) was suspended into 9 ml of modified tryptone soya broth. Samples were vortexed and incubated overnight at 41 °C. After selective enrichment, fifty micro liters of product was streaked onto Sorbitol MacConkey (SMAC) agar (Oxoid) supplemented with cefixime and the inoculated plates were incubated at 37 °C for 24 h. Then, up to six colourless colonies (non- Sorbitol fermenters) on SMAC agar were picked and separately sub-cultured on MacConkey agar (Oxoid) and incubated for 24 h at 37 °C for purification.

The purified and intensely red colonies with a pale periphery were tested for hydrogensulphide and indole production using Triple Sugar Iron agar (TSI) slant (Oxoid) and Indole production test. Those isolates giving a result of yellow slant and butt with gas but no hydrogen sulfide (Y/Y/ H_2_S -) production on TSI slant agar after incubation of the media at 37 °C for 24 h were kept with tubes capped loosely to maintain aerobic conditions. Indole test was carried out as follows. One pure colony was inoculated into 4 ml of tryptone soya broth (Oxoid) using a straight inoculation wire. Incubation was done for overnight at 37 °C. Then one drop of Indole (Kovac’s) reagent was added to the tryptone soya broth culture to test for indole production (formation of red ring indicating positive reaction).

The carcass bacterial swabs were incubated overnight at 41 °C after being suspended into modified tryptone soya broth (Oxoid) (1:9) and were subjected to tests for bacteriological analysis similar to the fecal samples.

Meat samples from each butcher shop were collected using universal bottle and 25 g of the meat was weighed, chopped aseptically and mixed with 225 ml of buffered peptone water; in a sterile plastic bag and homogenized using a homogenizer (stomacher 400, Seward Medical, England) at high speed for 2 min. The homogenate was then incubated at 41 °C for 16 to 18 h and subjected to similar tests for bacteriological analysis as fecal samples.

Environmental samples were incubated overnight at 41 °C after being suspended into modified tryptone soya broth (Oxoid) (at 1:9ratios) and subjected to similar tests for bacteriological analysis as fecal samples.

### Identification of *E. coli* O157:H7

Identification and confirmation of *E. coli* O157:H7was done at the Ethiopian Biodiversity Institute of (EBI), Addis Ababa, Ethiopia. Biochemically positive sample for *E.coli*were seeded onto nutrient agar and transported to the microbiology laboratory of EBI, using icebox. Upon arrival, the isolates were stored in a refrigerator at 4 °C. After preparing the rainbow agar O157 (Hayward, USA), the isolated colony from nutrient broth was inoculated by spreading suspected colonies of *E.coli* O157:H7on its surface. The plates were then incubated for 20 to 24 h, or longer, at 37 °C and observed for the presence of typical black or gray coloration on Rainbow agar O157 which shows pure colonies. The pure colonies were inoculated on BUG (Biolog Universal Growth Medium) agar (Hayward, USA) with 5% sheep blood and incubated at 37 °C for 24 h. Subculture was made using the same culture media to have pure culture colonies before identification was done by OmniLog as follows. First, bacterial suspension was prepared with an appropriate level of bacterial density, as recommended in the protocol of the instrument. Then the bacterial suspension was inoculated into the GEN III Micro Plates aseptically. The MicroPlates were covered with lid and incubated at 37 °C for 22 h. Then the Micro Plates were loaded into the OmniLog incubator/reader. The bacterial suspension was identified by the instrument using the inbuilt database.

### Antimicrobial susceptibility testing

The antimicrobial susceptibility test was performed following the standard agar disk diffusion method using commercial antimicrobial disks [27]. The selection criteria of the antibiotics depended on the regular use of the antimicrobials in the ruminants, potential public health importance and recommended from the guideline of antimicrobial susceptibility testing [27]. A standard reference strain of *E. coli* (ATCC 25922), sensitive to all antimicrobial drugs being tested, was used as a control.
